# The prevalence rate of sexual violence worldwide: a trend analysis

**DOI:** 10.1186/s12889-020-09926-5

**Published:** 2020-11-30

**Authors:** Nasrin Borumandnia, Naghmeh Khadembashi, Mohammad Tabatabaei, Hamid Alavi Majd

**Affiliations:** 1 Biostatistics, Urology and Nephrology Research Centre, Shahid Beheshti University of Medical Sciences, Tehran, Iran; 2 English Language Department, School of Allied Medical Sciences, Shahid Beheshti University of Medical Sciences, Tehran, Iran; 3 Medical Informatics Department, School of Medicine, Mashhad University of Medical Sciences, Mashhad, Iran; 4 Imam Reza Hospital Clinical Research Unit, Mashhad University of Medical Sciences, Mashhad, Iran; 5 Psychiatry and Behavioural Sciences Research Centre, Mashhad University of Medical Sciences, Mashhad, Iran; 6 Department of Biostatistics, School of Allied Medical Sciences, Shahid Beheshti University of Medical Sciences, Tehran, Iran

**Keywords:** Sexual violence, GBD study, Trend analysis

## Abstract

**Background:**

The purpose of the present study is to showcase the image of Sexual Violence (SV) temporal trends through exploring differences in its prevalence rates during 1990–2017 across 195 countries and territories.

**Methods:**

The SV prevalence rates were derived from the Global Burden of Disease (GBD) database during 1990–2017, worldwide. First, the Latent Growth Model (LGM) was employed for assessing the change in SV prevalence rate over time in Asia, Africa, Europe, North America, South America, Australia & Oceania, for men and women separately. Then, the change in SV prevalence rate over time was determined within countries with high and low Human Development Index (HDI). Finally, the Latent Growth Mixture Models (LGMM) were applied for identifying classes where countries within each class have similar trend of SV prevalence rate over time.

**Results:**

The SV prevalence was higher among women than men and decreased in both genders over time across the world. The declining trend in SV prevalence against men is visible in both countries with high and low HDI, but SV prevalence against women in countries with low HDI shows an increase. The findings of LGMM identified six classes of SV prevalence trajectories. LGMM allocated Bermuda into the class with the highest decrease in SV prevalence against men, and Equatorial Guinea and Luxembourg into the class with the highest increase. Other countries had very slow declining trends. In terms of SV prevalence against women, LGMM allocated China, North Korea, and Taiwan into the class with the most increase among the countries in the world. Bermuda, Guyana, Mexico, Nigeria, and Saint Lucia were placed into the class which witnessed the largest decline and Angola, Congo, and Equatorial Guinea were ranked next. The trend in other countries was mostly decreasing.

**Conclusion:**

Given the high economic and social burden that SV has on victims and societies, the rate of SV in most countries does not seem to have dropped remarkably and requires special attention by relevant policymakers. The SV prevalence rate is highly heterogeneous among world countries which may be due to the definitions and tools used, and more importantly, the culture norms.

## Background

Sexual Violence (SV) is defined as any sexual act or any attempt for the purpose of obtaining a sexual act through violence or coercion, which, according to WHO, encompasses a variety of situations, namely rape or marital infidelity, rape by strangers, sexual abuse, sexual or physical abuse of those with disabilities, sexual abuse of children, forced marriage and child marriage, denial of the right to use contraceptive equipment or prevention of sexually transmitted diseases as well as forced abortion [1]. Globally, about 35.6% of women have experienced SV, with widely varying prevalence estimates [2]. Men can also be subjected to SV, while it may be impossible to provide general prevalence rates, as SV is generally under-reported, with an elevated amount of non-reporting in case of violence against men and boys [3]. Most studies on SV among men have examined the above index in affected communities and war-torn areas [4].

Numerous studies have examined the prevalence of SV in different parts of the world on a cross-sectional basis in specific sub-communities, emphasizing women. An example would be studies over SV in children with mental disabilities (13.7%) [5], and violence in university on campuses [6]. The prevalence rate for completed sexual assault of undergraduates was 10.3% in women and 3.1% in men, and the prevalence rate for completed rape among undergraduates was 4.1% for women and 0.8% for men [7]. This rate against people with disabilities, such as mental disability was 5.5% [8], and 14.1% against older people [9]. Based on the systematic review by Araujo JO et al. in 2019, the prevalence of SV among refugees around the world was largely variable from 0 to 99.8%: in Africa, it has been reported from 1.3 to 99.8%, in Asia, the prevalence is variable from 0 to 84.6%, and in America and Europe, it has been reported 3.5% and 3.3, respectively. So, the prevalence of SV among refugees proved to be very different and expansive [10].

As seen in a few examples in the previous paragraph, various articles have studied SV prevalence in specific sub-communities. In addition, according to our literature review, all studies conducted on SV prevalence rates are mainly descriptive or cross-sectional and there are few studies for clustering countries by prevalence rates during the time. Hence, this study has a comprehensive look at the changes in the prevalence of age-standardized SV across all world regions, including 195 countries and geographical areas, from 1990 to 2017. To obtain complete information over the prevalence of SV in all regions of the world, the information provided by Global Burden of Diseases (GBD) study was used. This study was performed separately on men and women in various regions. Countries with high and low human development indicators are also compared in this regard. Moreover, countries worldwide are examined and categorized based on the rate of change in the prevalence of SV over the years. In this way, the areas where the prevalence of SV has witnessed a significant increase or decrease were identified, and the similarity of the trend of SV rate changes in countries were determined.

## Method

### GBD database

Data for the prevalence of SV (rates in 100,000 persons) in 195 countries and regions were derived from the GBD study related to Health Metrics and Evaluation (IHME). GBD exists as the most all-inclusive universal observational epidemiological study so far, with the power to describe mortality and morbidity (in terms of prevalence, incidence, disability-adjusted life-years (DALYs), death, etc. from major diseases, injuries, and risk factors to health at global, national, regional and country levels [11]. Data sources utilized in GBD take many forms, such as disease registries, surveys, scientific literature, population censuses, vital registrations and other large and small sources. In areas of the world with a lack of consistent and comprehensive censuses and vital registration systems, GBD uses some uncommon data sources. For example, verbal autopsy data through the trained interviewers may be used in these situations. More information about the GBD database can be reached through the mentioned reference [12]. The extracted information includes prevalence rates of SV for men and women, from 1990 to 2017. At first, six regions were considered, including Asia, Africa, North American, South America, Europe, and Australia & Oceania. These regions include all of the countries designated by the GBD Study. The trend of prevalence rates of SV in each mentioned region was estimated separately for men and women. All countries were then categorized into a development status according to HUMAN DEVELOPMENT REPORT of 2019 [13]. The Human Development Index (HDI) is a summary measure of achievements in three critical dimensions of human development: a long and healthy life, access to knowledge, and a decent standard of living [13]. The trend of prevalence rates of SV among countries with high HDI (> = 0.7) and also among countries with low HDI (< 0.7) were explored separately for men and women. Finally, all countries were explored in terms of their trends of SV over the years.

### Statistical method

In the descriptive statistics section, SV prevalence in each region and year was reported with mean and standard deviation. The Latent Growth model (LGM) and Latent Growth Mixture model (LGMM) were applied. The LGM methods estimated the outcome growth trajectory over time. LGM can be extended to the LGMM to taking into account heterogeneity in growth trajectories. LGMM is often used to determine if subgroups exist within the population that follow similar trends over time. In other words, LGMM represents subpopulations where population membership is not known but is inferred from the data [14]. Hence, the LGM has been used to examine the trend of changes in the prevalence of SV in different continents, separately for men and women, as well as in developed and developing countries. For LGM analysis in this study, the continuous response variable is SV prevalence in each country from 1993 to 2017. The coefficients of these models are interoperated as the average rate of outcome changes over time. Given that each country has a different trend over time, the LGMM analysis method was employed. The LGMM method, generalized from LGM was used to categorize the world’s countries in terms of how their prevalence of SV has changed over time. In this way, it can be specified which countries have had a similar trend. LGMM determines the categories of countries based on two criteria: the similar trend of SV prevalence rate and the similar onset of the SV prevalence rate at the beginning of study period. The interpretation of the results in this model is similar to the LGM. Statistical analysis was done using M-plus software, version 6.12 (www.statmodel.com). The R 3.6.2 software with “rworldmap” package was used for mapping global data.

## Results

The descriptive statistics, including mean (SD) of SV rate as well as estimates from the LGM are shown in Table 1, separately for region and gender. The crude SV prevalence rates in the table revealed that Australia (for men and women) had the highest and Europe (for men and women) and South America (for men) had the lowest SV prevalence rates during these years. In all regions, the crude SV prevalence rates were higher in women than men.

**Table 1:** Prevalence rates (per 100,000 persons) of sexual violence as mean (SD) and estimates from the LGM by the regions for trend analysis. The intercepts represent the estimated overall mean level of the initial sexual violence rate and the slopes show the average rate of change in sexual violence rate over time within each region

Region	Gender	Years					LGM estimates	*p*-value
		1993	1999	2005	2011	2017		
Asia	Male	1122.7 (393.8)	1116.9 (385.3)	1116.2 (389.2)	1110.6 (372.5)	1109.9 (365.1)	Intercept:1117.89	< 0.001
							Slop: −1.66	0.506
	Female	2442.8 (2269)	2426.5 (2245.3)	2445 (2294.8)	2402.2 (2169.9)	2424.3 (2217.4)	Intercept:2449.1	< 0.001
							Slop: −5.6	0.467
Africa	Male	1421.8 (412.3)	1397.8 (379.5)	1431.8 (432.2)	1431.2 (423.2)	1415.3 (383.4)	Intercept:1421.93	< 0.001
							Slop: 3.07	0.660
	Female	3350.1 (1653.7)	3342.9 (1598.7)	3343.4 (1637.9)	3410.7 (1810)	3402.2 (1762.2)	Intercept:3344.9	< 0.001
							Slop: −1.4	0.608
Europe	Male	981 (279.3)	935.7 (224.3)	921.3 (207.8)	913.5 (199.4)	908.7 (183.5)	Intercept:927.57	< 0.001
							Slop: −3.65	0.455
	Female	1837.4 (548.7)	1764 (433.8)	1805.8 (505.8)	1742.4 (402.1)	1737.8 (410.4)	Intercept: 1709.3	< 0.001
							Slop: −4.99	0.624
North America	Male	1065.8 (471.7)	1047.4 (460.1)	1026.4 (407.4)	1009.4 (343.6)	1002.1 (316.6)	Intercept:1062.82	< 0.001
							Slop: −16.97	0.169
	Female	3117.8 (1156.1)	3004.3 (1124.5)	3094 (1158.2)	2822.8 (876.3)	2821.4 (811.2)	Intercept:3175.5	< 0.001
							Slop: −90.1	< 0.001
South America	Male	1008.3 (253.3)	971.3 (235.2)	925.4 (208.2)	910 (190.5)	930.4 (172.1)	Intercept:999.47	< 0.001
							Slop: −30.2	0.590
	Female	2635.8 (731)	2499.3 (694.7)	2617.9 (729.1)	2364.6 (675.4)	2349.7 (670.7)	Intercept:2531.6	< 0.001
							Slop: −32.12	0.223
Australia & Oceania	Male	1475.7 (383.7)	1471.8 (383.5)	1468.1 (381.1)	1471.5 (375.8)	1478.4 (371.1)	Intercept:1471.65	< 0.001
							Slop: −0.02	0.997
	Female	4410.3 (1165.3)	4380 (1257.4)	4404.3 (1187.5)	4371.5 (1245.5)	4357.5 (1260.5)	Intercept:4386.8	< 0.001
							Slop: −5.2	0.593
Global^a^	Male	1193.9 (423.8)	1164.5 (405)	1166.2 (420.1)	1160 (407.1)	1155.3 (186.8)	Intercept:1177.7	< 0.001
							Slop: −1.6	0.128
	Female	2816.1 (1704.5)	2769.6 (1698.3)	2803.8 (1709.8)	2744 (1703.3)	2745 (1699.6)	Intercept:2788.9	< 0.001
							Slop: −2.1	0.013

^a^The LGM results are related to the prevalence rates of sexual violence, repeated in every 2 years, from 1990 to 2017. In the others, the rates repeated in every 6 years, from 1993 to 2017, due to the small sample size consideration.

In the LGM estimates column of Table 1, both the estimated intercepts and slopes can help us know more about SV prevalence trends in these regions. The intercepts represent the estimated overall mean level of the initial SV prevalence rate and the slopes show the average rate of change in SV prevalence rate over time within each region. A positive and negative slope reveals that the rate had an incremental and decremental trend over time, respectively.

For instance, the estimates for men in Asia (Intercept = 1117.8, Slope = − 1.6) state that the initial SV prevalence rate in this region was 1117.8 in 100,000 persons in 1993, and the prevalence rate has a decremental trend with a slope of 1.6 during 1993 to 2017 in every six-year period. Regarding the estimated slope in Table 1, with the exception of African men, SV prevalence rate has declined across all continents and in both sexes. The most decline is witnessed in the SV prevalence rate among North American women (rate of 90.1 per 100,000 persons), followed by women in South America (rate of 32.12 per 100,000 persons). Also, the lowest decline was in the prevalence of SV among Australian men (rate of 0.02 per 100,000 persons).

The last row in Table 1 gives us global information about the intercept and trend of SV prevalence rate, separately for men and women. Regarding this, one can conclude that SV prevalence rate in women was greater than men (2816.1 vs 1193.9 per 100,000 persons). In addition, the trend of SV prevalence rate in women was more decreased than men (2.1 vs 1.6 per 100,000 persons).

As shown in Table 1, except for North America and Global (for women), the trend coefficient (slope) has not been significant in other regions (*p*-value> 0.05). As can be seen, the coefficient of − 2.1, for women in global, is significant, while the coefficient of − 32.12 is not significant for South America women. The most important reason for the lack of significance is the small number of countries in the regions, and is not necessarily the reason for insignificance of the coefficient value.

Subsequently, countries were classified according to the HDI; countries with high HDI (> = 0.7), and also countries with low HDI (< 0.7) [13]. The descriptive statistics, and estimates from the LGM, separately for countries with high and low HDI, are shown in Table 2. The crude SV prevalence rates in Table 2 indicates that countries with high HDI (for men and women) had the higher SV prevalence rates compared with countries with low HDI during these years. Totally, the crude SV prevalence rates were higher in women in both high- and low-income countries. The LGM estimate column in Table 2 shows that the declining trend in men’s SV is visible in both high and low HDI countries. Also, it shows the prevalence of SV against women in countries with low HDI has increased (average rate of 5.1 per 100,000 persons).

**Table 2:** Prevalence rates (per 100,000 persons) of sexual violence as mean (SD) and estimates from the LGM by the HDI for trend analysis. The intercepts represent the estimated overall mean level of the initial sexual violence rate and the slopes show the average rate of change in sexual violence rate over time within each region

	Gender	Years					LGM estimates^a^	*p*-value
		1993	1999	2005	2011	2017		
Human Development > = 0.7	Male	1130 (366.5)	1114.9 (369.8)	1107.6 (375.1)	1102.2 (367.7)	1097.8 (341.5)	Intercept:1121.1	< 0.001
							Slope: −6.24	0.136
	Female	2678.6 (1693.7)	2634.9 (1690)	2665.9 (1707.3)	2612.1 (1722.8)	2602.1 (1709.6)	Intercept:2672.5	< 0.001
							Slope: − 0.7	0.667
Human Development < 0.7	Male	1258.7 (461.7)	1243.9 (454.8)	1236 (440.4)	1226.6 (419.9)	1221.2 (405.7)	Intercept:1252.9	< 0.001
							Slope: −8.58	0.205
	Female	2973.2 (1699.2)	2933.8 (1663.4)	2963.8 (1695.3)	2900.3 (1661.9)	2915.1 (1670.4)	Intercept:2934	< 0.001
							Slope:5.1	0.747

^a^The LGM results is related to prevalence rates of sexual violence, repeated in every 2 years, from 1993 to 2017

**Fig. 1:**
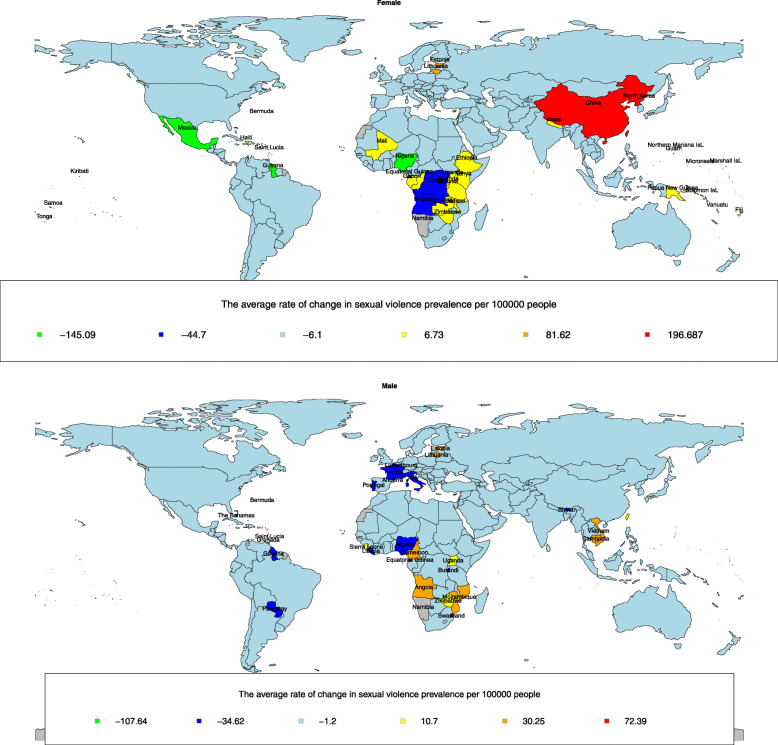
World cluster map on the basis of sexual violence’ outbreak trends within the years 1993 to 2017. This map shows the result of the latent growth mixture model in our own study

Finally, using LGMM, countries around the world were categorized separately according to the trend of SV rate changes, for men and women. According to these results, countries were classified into six classes with various SV prevalence trends. In other words, the countries with similar trend has been placed in the same class. These classes are presented using zoning maps in Fig. 1. In this figure, countries with similar colors on the map have had similar trends of SV prevalence and have been included within the same class. The map is our own result and is not taken from any other source. As shown in the zoning map of SV against men, the highest decrease in the prevalence of SV was in Bermuda (rate of 107.64 per 100,000 persons). Andorra, Bhutan, Burundi, France, Grenada, Guyana, Italy, Liberia, Nigeria, Paraguay, Portugal, Saint Lucia, Swaziland and Bahamas had also a decreasing trend (average rate of 34.62 per 100,000 persons). The highest increase in the prevalence of men’s SV was also observed in Equatorial Guinea and Luxembourg (average rate of 72.39 per 100,000 persons). Also, Angola, Cambodia, Cameroon, Estonia, Lithuania, Mozambique, Namibia and Vietnam have been ranked next in terms of increasing trend in SV prevalence rate (average rate of 30.25 per 100,000 persons). The aforementioned countries had distinct trends from others, while most of other countries had very slow declining trends (average rate of 1.2 per 100,000 persons).

Considering the map of SV against women, countries with a distinct trend are as follows: SV against women in China, North Korea, and Taiwan has increased the most among countries in the world (average rate of 196.68 per 100,000 persons). Lithuania and Namibia have been ranked next (average rate of 81.62 per 100,000 persons). In addition, the map shows Bermuda, Guyana, Mexico, Nigeria, and Saint Lucia have witnessed the largest declines in the prevalence of women’s SV during the time of study (average rate of 145.09 per 100,000 persons). Angola, the Democratic Republic of Congo, and Equatorial Guinea have been ranked next (average rate of 44.70 per 100,000 persons). The observed trend in terms of changes in the prevalence rate of SV against women in other countries has been mostly decreasing (average rate of 6.10 per 100,000 persons).

## Discussion

Sexual violence is a pertinent health challenge, which has increased the risk of various sexual and reproductive health problems and impacts physical and mental health [1]. According to our literature review, all studies conducted on SV prevalence rates are mainly descriptive or cross-sectional in sub-population and changes over time are not well documented. Hence, the present study assessed the trends of SV prevalence rates across all countries in 25 years from 1993 to 2017 to make comparisons across six GBD regions.

Results show that globally, SV prevalence rate against women are higher than men. SV against women, influenced by cultural factors and values is often a result of unequal power equations between men and women [15]. By contrast, SV against men is less understood or acknowledged. It seems that because men are the stronger sex, they have a lower rate of SV than women. Also, based on gender norms combined with cultural and religious taboos and scarce services, it may be challenging for men to disclose that they are survivors of SV. In addition, service providers may not recognize the male experience of SV. Therefore, in such societies with gender norms combined with cultural and religious taboos, SV prevalence rate in men may be underestimated [3].

The present study results also manifested that the decremental trend of SV prevalence in Europe and the United States are more outstanding than in other regions. This is particularly prominent in the Americas, where there has been a decline in SV prevalence in both sexes, with the largest decrease in the prevalence of SV against women. In Asia as well as Australia, this declining trend has occurred, albeit to a lesser extent, compared to the above continents. It is vital to point that, although the prevalence rate of SV against women has decreased to some extent in African countries, it has increased against men. Civilians in Africa’s conflict zones, both women and men, are often vulnerable to SV, and most of the SV in these areas is due to this fact [16]. Of course, it should be kept in mind that the rate of low SV against women in some societies might be related to victimized women being unlikely to report an attack due to fear of discrimination, feeling shame, and not being able to identify [17].

Further, the results of this study show that SV prevalence rate is almost the same in both countries with high and low HDI. Also, results showed the prevalence rate of SV in countries with high HDI has been declining, and in countries with low HDI, it has been declining against men and increasing against women in recent years. It should be pointed that in countries with high HDI, there are effective programs, for example the ‘Universities Supporting Victims of Sexual Violence: Training for Sustainable Student Services project, running across seven European countries, which help university staff respond more effectively to disclosures of SV [18]. Moreover, countries with high HDI are usually high income, and the studies show that estimates over SV from high-income countries seem to be lower than those from low and middle-income countries [17]. Most countries with a low HDI are cultural communities where human relationships are at the core, and individual identity is subsumed in the family or kinship, causing gender bias and blaming of the victim. Therefore, such cases are often not reported [19]. Hence, the lack of reporting of these cases in such societies may have caused SV prevalence rate in countries with high and low HDI to be reported as close.

The paper examined which countries in the world have experienced a similar trend of SV prevalence rate in recent decades. The results manifested that, among the countries of the world, the highest decline in the prevalence of SV against men occurred in Bermuda. Similarly, the results showed that SV against women has been declining in some African and American countries such as Bermuda, Guyana, Mexico, Nigeria, and Saint Lucia. The Democratic Republic of Congo, and Equatorial Guinea have been ranked next. Programs aimed at reducing SV among such communities may have been effective in reducing the rate of SV. Based on available studies, it appears that the most activity is in some African countries [20].

Also, the LGMM results showed the highest increase in prevalence rates of SV against men was in Luxembourg and Equatorial Guinea, and as for women, it was reported in China, North Korea, and Taiwan. The high prevalence of SV against women in china has been estimated in recent studies which are in line with the present study’s results and the recent announcement of the Domestic Violence Law represents a first step to raise awareness and prevention of violence against women [21, 22]. No study with a candid look at North Korean women was found, and yet several studies have examined SV between undocumented refugees without basic legal protections, and subsequently they become exposed to human trafficking, with sex trade being built upon their exploitation [23].

This study embodies some limitations. Lack of data for SV prevalence rate in some countries at a specific time makes GBD report the estimated rates. Another limitation of the present study is that the latest information provided in the GBD Database is related to 2017, and the information for 2018 to 2020 has not been published yet.

## Conclusion

This study highlights the rate of worldwide SV prevalence and concludes it to be highly heterogeneous. This may be due to the definitions and tools used, and more importantly, due to the culture and norms. Study findings also underline that the SV rate in most countries does not seem to have dropped remarkably and requires special attention by relevant policymakers. Given the high economic and social burden that SV has on victims and society, it is emphasized that programs on sexual and gender-based violence, including men as well as women, provide guidance on how to access survivors, facilitate reporting, provide protection and deliver essential medical, legal and social services. Considering that there are gaps in understanding who the victim of SV is in different cultural contexts and societies where the notion of the dominance of men over women prevails, implementation of national population prevalence surveys is necessary for all countries for establishing SV as a globally severe social issue.

## Data Availability

The datasets used and analyzed during the current study is available from IHME at: http://ghdx.healthdata.org/gbd-results-tool.

## Abbreviations

SV: Sexual Violence
GBD: Global Burden of Disease
LGM: Latent Growth Models
LGMM: Latent Growth Mixture models
HDI: Human Development Index
